# Editorial: Breathing in sport and exercise: physiology, pathophysiology and applications

**DOI:** 10.3389/fphys.2023.1347806

**Published:** 2023-12-12

**Authors:** Andrea Nicolò, Mathieu Gruet, Massimo Sacchetti

**Affiliations:** ^1^ Laboratory of Exercise Physiology, Department of Movement, Human and Health Sciences, University of Rome “Foro Italico”, Rome, Italy; ^2^ Université de Toulon, J-AP2S, Toulon, France

**Keywords:** respiratory frequency, tidal volume, ventilatory control, breathing strategies, incremental exercise, technology, breath holding, respiratory muscle training

## 1 Introduction

The purpose of this Research Topic was to improve our understanding of the physiology and pathophysiology of breathing during exercise and exploit this knowledge to advance the field of breathing monitoring in applied settings. The importance of breathing monitoring is currently underappreciated in the field of sport and exercise, partially because the pulmonary system has long been considered overbuilt for exercise (Dempsey et al., 2020; Peters et al., 2023). However, the interest in breathing monitoring during exercise is growing progressively for a number of reasons: 1) the ventilatory variables [especially respiratory frequency (*f*_R_)] are particularly sensitive to changes in exercise tolerance; 2) *f*_R_ and tidal volume (V_T_) are to a large extent modulated by different inputs during exercise, and their separate measurement provides relevant information (Nicolò and Sacchetti, 2023); 3) an abundance of technological solutions is currently available to monitor breathing variables in applied settings (Massaroni et al., 2019); and 4) different breathing strategies can be implemented for research or applied purposes. Most of these points have been explored by the 16 articles published in this Research Topic. We have grouped the articles into three different categories, i.e., physiology, pathophysiology and applications, but, as intended in the conception of the Research Topic, many of them show some overlap with other categories.

## 2 Physiology

Two studies focused on the mechanisms underlying ventilatory control during exercise. The fact that *f*_R_ is sensitive to changes in exercise tolerance and is largely modulated by different inputs compared to V_T_ is corroborated by findings from Nicolò et al. They found that *f*_R_–but not oxygen uptake and heart rate–was sensitive to the improvement in exercise tolerance observed when comparing arm + leg cycling with conventional leg cycling. This response was observed both during incremental and time-to-exhaustion tests, and a high sensitivity to improvements in exercise tolerance was also found for perceived exertion. These findings support the notion that *f*_R_ is a marker of physical effort and is substantially modulated by central command during high-intensity exercise (Nicolò and Sacchetti, 2023). A degree of interindividual variability in the breathing pattern was observed in this study, although the breathing pattern appeared to be generally consistent across tests for the same individual. To shed some light on the interindividual differences in breathing responses observed during incremental exercise, Harrison et al. evaluated whether ventilatory and anxiety responses during exercise are associated with ventilatory and anxiety responses to hypercapnia at rest. They found that this association may be moderated by the fitness level, as a positive association was found in healthy untrained individuals but not in endurance-trained athletes. Besides, higher V_T_ values and lower *f*_R_ values were found in athletes during hypercapnia, despite similar minute ventilation values. Future studies should further our understanding of the mechanisms underlying interindividual differences in breathing patterns during exercise.

Different studies focused on the effect of voluntarily modulating the breathing pattern, and two of them investigated the effects of breath-holding. This intervention induces a variety of physiological responses that are supposed to determine performance benefits. Yang et al. tested the effect of 8 weeks of dry dynamic breath-hold training on spleen volume and hematological parameters. Performing breath-holding during exercise was supposed to elicit a greater stimulus compared to static breath-holding. This intervention was sufficient to improve apnoea tolerance and increase spleen volume, while no increase was observed in red blood cell and hemoglobin levels. Breath-holding is also included in popular breathing techniques such as the Wim Hof Breathing Method (WHBM), which combines periods of hyperventilation followed by voluntary breath-holds at low lung volume. The acute effect of this method on repeated sprint ability was investigated by Citherlet et al. and compared with a condition where only hyperventilation was performed. While hyperventilation induces respiratory alkalosis, breath-holding counteracts the increase in blood pH and triggers the so-called diving response, leading to a series of acute physiological responses including spleen contraction. However, none of the two interventions managed to improve repeated sprint performance. Furthermore, some participants reported side effects in the hyperventilation and WHBM conditions, including dizziness, heaviness or deafness. Hence, caution is suggested when attempting to implement breath-holding strategies into training as they may not provide performance benefits or may even result in side effects.

Side effects may also be experienced when performing saturation diving, and Lian et al. investigated the effects of a single simulated 500-m saturation dive on the lung function of professional divers. They found a decrease in small airway function and diffusion function as assessed by means of pulmonary function tests performed before the dive and 3 days after. They also found that divers showing small airway dysfunction or mild obstructive lung function defects before the dive might be at greater risk of developing further post-dive abnormalities. This outlines the importance of an individualized approach to health assessment and management in this population.

## 3 Pathophysiology

Breathing monitoring has important implications for the identification of pathophysiological signs of cardiopulmonary and metabolic diseases. Hyrylä et al. focused on the “diabetic lung”, which is an overlooked target organ of the disease (Pitocco et al., 2012). As the impairment in pulmonary function may not be evident in the first stages of diabetes, the authors attempted to identify early signs of deterioration by analyzing the morphology of the flow signal within the respiratory cycle during a cardiopulmonary exercise test (CPET). They found significant differences between healthy individuals and well-controlled type I diabetic males, the latter showing an attenuated expiratory slope (from expiratory onset to expiratory peak flow) at peak exercise but not during submaximal exercise. While the preliminary nature of the study does not allow us to speculate on the clinical relevance of such findings, a careful examination of the morphology of the breathing signal may complement more traditional assessments performed with a CPET. For instance, a CPET can be used to monitor cardiorespiratory function after recovering from surgeries or infections, as done by Lemos et al., who tested 171 overweight or obese individuals who had contracted COVID-19. They found a greater exercise tolerance in non-hospitalized patients than in patients who were hospitalized or admitted to the intensive care unit. Furthermore, non-hospitalized patients showed a higher peak oxygen uptake and an improved recovery of vital signs after the incremental test. The exploitation of the full potential of CPET is encouraged in people with cardiopulmonary and metabolic diseases.

Technological advances favor breathing monitoring outside the laboratory, with important implications for the identification of adverse events in daily life. Wang et al. monitored 578 people with respiratory disorders performing the 6-min walk test while wearing a flexible vest recording electrocardiographic, breathing and accelerometric signals, and oxygen saturation and blood pressure values. The purpose of the study was to develop models predicting adverse events occurring during the test, combining vital sign monitoring with demographic data and artificial intelligence. The models developed have the potential to provide assistive decision support for identifying patients who may require medical help during the test. This is an example of how technology may favor ubiquitous breathing monitoring in daily life and help tailor therapies to individual needs and assess their effects. Indeed, some breathing variables are sensitive to clinical deterioration, pain and infections (Nicolò et al., 2020), and the breathing signal may also be used to retrieve other useful information (e.g., number of cough events) (Otoshi et al., 2021). The impact of inhaled corticosteroids on the cough reflex was investigated by Basin et al., who used an animal model to address this issue. They found restoration of the desensitization of the cough reflex during exercise when inhaling corticosteroids, and airway inflammation appeared to be involved in the modulation of this response. These findings suggest that anti-inflammatory treatments may help manage exercise-induced cough in people with asthma.

## 4 Applications

We live in a time where the respiratory system has become a target of the multi-trillion-dollar health and wellness industry (Illidi et al., 2023). There is an abundance of interventions that are purported to provide benefits to the respiratory system, but commercial claims are rarely supported by scientific evidence. Some studies have attempted to shed some light on this issue, starting with the work by Harbour et al., who have reviewed the available literature on the advantages and disadvantages of adopting breathing strategies that may potentially enhance running performance. They also pointed toward the importance of using technology to help runners learn such breathing techniques. Among a variety of strategies reviewed, they focused on nasal breathing and respiratory muscle training, which have also been investigated by other articles published in this Research Topic.

Nasal breathing has received attention as it stimulates the endogenous production of nitric oxide, which being a vasodilator and a mild bronchodilator favors blood flow redistribution in the different lung regions, among other effects (Illidi et al., 2023). Nasal breathing during exercise requires awareness and training, but the feasibility of this strategy improves after some months of practice, even at relatively high exercise intensities (Harbour et al.). While the benefits of nasal breathing in exercising healthy individuals still need to be proven satisfactorily, Rappelt et al. investigated the acute effects of restricted nasal-only breathing on self-selected exercise intensity in a 60-min exercise session. They hypothesized that nasal breathing would help the participants perform low-intensity endurance training at the required intensity instead of selecting higher intensities than intended. However, no significant differences were observed in power output distribution between the nasal-only condition and the oro-nasal condition, but a lower *f*_R_ was found in the former condition. A lower *f*_R_ and a higher V_T_ were also found by Held et al. when comparing high-intensity exercise performed in the uphill running modality vs level running modality. While nasal-only breathing and uphill running may be useful modalities for investigating the control of *f*_R_ and V_T_ during exercise, the two studies were not designed with this intent, hence requiring further investigation.

When prescribed with the appropriate intensity, duration and frequency, respiratory muscle training appears to be effective in improving respiratory muscle strength/endurance and exercise capacity in healthy individuals (Illidi et al., 2023). Shei et al. outlined the importance of individualizing the prescription of inspiratory muscle training, while many of the studies conducted so far have used fixed protocols, some of which were developed for clinical populations. As it is commonly done for the locomotor muscles, the respiratory muscles could be trained by considering the principles of training, including individualization, periodization and specificity. For instance, the authors suggest that the intensity selection may take into account the breathing pattern and the demands of exercise. The reliability of the tests designed to measure important functional outcomes of the respiratory muscles (e.g., endurance) should also be considered when interpreting the efficacy of a respiratory muscle intervention (Larribaut et al., 2020). By prescribing respiratory muscle training according to the ventilatory demands of actual exercise, Chambault et al. found a greater improvement in cycling time trial performance in women than in men, and this effect was more pronounced in hypoxia. These findings are in line with evidence suggesting that the pulmonary system is challenged during exercise more in women than in men, and even more in hypoxic environments (Dempsey et al., 2020). This reinforces the suggestion by Shei et al. that respiratory muscle training should be tailored according to individual needs.

Another topic that has recently regained attention is the impact of apparatuses or protective devices used in occupational settings on breathing and related perceptions. The wide use of face masks during the COVID-19 pandemic has triggered interest in understanding their effect on exercising humans. Glänzel et al. provided a meta-analysis of the acute effects of mask-wearing during exercise on performance and psychological responses. They observed a small reduction in performance during time-to-exhaustion tests but not during most of the other exercise tests used. However, they reported increases in discomfort (large effect), dyspnea (moderate effect) and perceived exertion (small effect), although some of these effects were dependent on the type of mask used. Rives et al. simulated the inspiratory load experienced by military divers wearing a rebreather device and were able to characterize the response of the diaphragm using ultrasound. The authors found an increase in both the excursion and the thickening fraction of the right hemidiaphragm and suggested the latter being a relevant parameter to be considered in inspiratory load evaluation. While apparatuses and protective devices used in occupational settings are sometimes considered detrimental to exercise capacity or comfort, they may instead serve as facilitating tools for vital sign monitoring and work management. Indeed, face masks or other protective devices can be equipped with sensors and used for providing services to the user (Li et al., 2023).

## 5 Conclusion

The studies published in this Research Topic support the importance of monitoring breathing for a variety of applications. A good understanding of the physiological and pathophysiological responses to exercise should guide the monitoring of breathing in applied settings. Furthermore, special attention should be given to interindividual differences in breathing responses to tailor breathing and exercise strategies to individual needs. We hope this Research Topic may have contributed to narrowing the gap between physiology, pathophysiology and applications in the context of breathing monitoring during exercise.
